# Integration of curated databases to identify genotype-phenotype associations

**DOI:** 10.1186/1471-2164-7-257

**Published:** 2006-10-12

**Authors:** Chern-Sing Goh, Tara A Gianoulis, Yang Liu, Jianrong Li, Alberto Paccanaro, Yves A Lussier, Mark Gerstein

**Affiliations:** 1 Molecular Biophysics and Biochemistry, Yale University, New Haven, USA; 2 Computer Science, Yale University, New Haven, USA; 3 Program in Computational Biology and Bioinformatics, Yale University, New Haven, USA; 4 Department of Biomedical Informatics, Columbia University, New York, USA; 5 Department of Computer Science, Royal Holloway University of London, Egham, UK

## Abstract

**Background:**

The ability to rapidly characterize an unknown microorganism is critical in both responding to infectious disease and biodefense. To do this, we need some way of anticipating an organism's phenotype based on the molecules encoded by its genome. However, the link between molecular composition (i.e. genotype) and phenotype for microbes is not obvious. While there have been several studies that address this challenge, none have yet proposed a large-scale method integrating curated biological information. Here we utilize a systematic approach to discover genotype-phenotype associations that combines phenotypic information from a biomedical informatics database, GIDEON, with the molecular information contained in National Center for Biotechnology Information's Clusters of Orthologous Groups database (NCBI COGs).

**Results:**

Integrating the information in the two databases, we are able to correlate the presence or absence of a given protein in a microbe with its phenotype as measured by certain morphological characteristics or survival in a particular growth media. With a 0.8 correlation score threshold, 66% of the associations found were confirmed by the literature and at a 0.9 correlation threshold, 86% were positively verified.

**Conclusion:**

Our results suggest possible phenotypic manifestations for proteins biochemically associated with sugar metabolism and electron transport. Moreover, we believe our approach can be extended to linking pathogenic phenotypes with functionally related proteins.

## Background

Traditionally, microbes have been identified on the basis of their response to a battery of phenotypic assays, for example, survival on a particular type of growth media or morphological characteristics. With the advent of high throughput sequencing efforts, over 300 microbes have been completely sequenced [1]. By integrating complex phenotypic data with sequence information, new phenotype-genotype relationships can be unveiled.

The underpinnings for this work can be found in Marcotte *et al*. where phenotype was defined in terms of pathway membership which was used to predict protein function [2]. In addition, previous studies have proposed comparative genomic methods to predict characteristics such as hyperthermophily [3,4], flagellar motility [4-6], plant degradation [6], and pili assembly [4]. However, most of these studies focus on a few specific phenotypes within certain organisms [3-5]. Korbel *et al*. proposed an automated method to make word-species associations retrieved from Medline abstracts [6]. Here we introduce a new approach for discovering phenotype-genotype relationships using a clinical information database consisting of manually curated results from 93 phenotypic assays allowing for a large-scale analysis of phenotype-genotype relationships.

The Global Infectious Diseases & Epidemiology Network (GIDEON) is an expert system used primarily by physicians to aid in the diagnosis of infectious diseases [7]. This database was chosen because of its exhaustive categorization of microorganisms. The results of 93 different microbiological assays for 1147 microbial taxa are catalogued providing a wealth of phenotypic data. GIDEON is manually annotated using peer-reviewed sources in the scientific literature.

NCBI's Cluster of Orthologous Groups of proteins (COGs) database currently consists of 138,458 proteins, which form 4873 COGs [8-10]. This database phylogenetically classifies proteins from complete genomes into COGs where each COG includes proteins that are thought to be orthologous. All the newly classified COGs and new members of pre-existing COGs are manually curated.

This study uses the COGs database and the GIDEON database by linking the proteins found in organisms (COGs) to the organism's expressed phenotype (GIDEON) Using this approach, we are able to identify phenotype-COG association pairs and verify these findings in the literature. Finally, this analysis suggests possible phenotype-genotype pairs that have not yet been experimentally determined. By integrating a clinical microbiological database, GIDEON, with a molecular database, COGs, we can make inferences between the presence of a protein and the protein's function in a large-scale fashion.

## Results

By utilizing the phenotypic information stored in the GIDEON database, we can begin to make associations between the presence of a gene (COG) within an organism to its expressed phenotype. First, we mapped the organisms found in the GIDEON database to the organisms in the COGs database (see Additional file 1). Secondly, to quantify the associations between the phenotype of an organism to its genomic content, we calculated the correlation between the measured expression of a certain phenotype to the absence or presence of COGs within an organism. Third, we applied a hypergeometric distribution threshold of 0.01 to filter for significant correlations.

Subsequently, a 0.8 correlation threshold and a 0.9 correlation threshold were used to generate two separate result sets. The 0.8 correlation data set contained 290 association pairs (see Additional file 2). One hundred random data points were selected from the 0.8 correlation result set for literature validation; these are referred to as annotated pairs (see Additional file 3). Out of these 100 data points, 66% of the data points had associations confirmed in the scientific literature. For the 0.9 correlation score threshold, 86% (31/36) of the resulting pairs had confirmed associations in the literature (Table 1).

Some of the representative association pairs that were verified by the literature are discussed below (Table 2). The laboratory conditions are referred to by their GIDEON identifier/phenotypic description (see Additional file 4), and a COG with a known function is defined as characterized (see Additional file 5).

A) B01/Gram-negative – Among the 66 confirmed associated pairs found in the result set with a threshold score of 0.8, 16 substantiated associations out of a total of 17 annotated COG-phenotype pairs (Table 2) are involved in the B01/Gram-negative phenotype. This resulted in 94% accuracy for determining Gram-negative organisms. For the data set with a score of 0.9 correlation criteria, 12 out of 12 total annotated COG-phenotype pairs were verified by the literature.

The outer membrane of the Gram-negative bacteria is a lipid-protein bilayer made up of proteins, phospholipids, and lipopolysaccharides that differentiate it from the thicker cell wall structure of Gram-positive bacteria [11]. Perhaps unsurprisingly, the confirmed pairs found with the Gram-negative phenotype contained proteins involved with lipid A and lipopolysaccharide biosynthesis and other proteins belonging to the outer membrane of Gram-negative bacteria (Table 3).

B) B02/Gram-positive – More interestingly, annotated pairs with the B02/Gram-positive phenotype were not just specific to the specialized Gram-positive membrane but also to a variety of conserved genes found only in the Gram-positive bacteria. Of the six proteins with known function found in the 0.8 correlation score data set, 3 pairs were positively confirmed by the scientific literature. In the 0.9 correlation result set, 2 out of 2 (100%) of the characterized pairs were corroborated.

C) B29/Growth on MacConkey Agar – Growth on MacConkey agar is indicative of Gram-negative bacteria that can ferment lactose [12]. Sixteen (52%) of the associated pairs from the 0.8 correlation data were confirmed, as were 4 (100%) of the associated pairs from the 0.9 correlation set Organisms that were able to grow on MacConkey agar contained proteins involved with the outer membrane of Gram-negative bacteria. Given the use of this test in specifically differentiating those Gram-negatives that can ferment lactose, one would expect this result; however, the proteins associated with growth on MacConkey agar do not overlap with those proteins most associated with the Gram-negative test. This suggests that this method of building associations can be specific to a particular condition.

D) B30/Oxidase – Two characterized COGs were found with a correlation at 0.85 and *p *< 7.48 × 10^-6 ^to be positively associated with oxidase activity. Both are components of Cbb3-type cytochrome oxidase which is unsurprising since the goal of the oxidase test is to detect the presence of this enzyme. Although this is not a novel finding, it does illustrate that our method is able to pick out known relationships.

E) B31/Catalase – In the catalase test, hydrogen peroxide is added to the media. Those microbes which do not contain the catalase enzyme are unable to break the hydrogen peroxide into oxygen and water and would die. As would be expected, the COGs associated to the B31/Catalase test were usually enzymes that belong to similar regulation pathways as the catalase enzyme. For example, human acyl-CoA hydrolase, one of the COGs found to be positively associated to the catalase phenotype, upregulates peroxisome biogenesis and, in turn, activates catalase activity [13]. The highest scoring pair was a member of the catalase protein family. For both the 0.8 and 0.9 correlation result sets, the confirmation percentages were 64% and 63% respectively.

F) FAC/L-Arabinose – With a high correlation score of 0.97, 5-keto 4-deoxyuronate isomerase was the only characterized protein family associated with the ability to assimilate arabinose. 5-keto 4-deoxyuronate isomerase, or kduI, is an enzyme involved with pectin degradation and shares the same regulator protein, crp or CAP protein, as the L-Arabinose catabolism pathway [14,15].

G) FAT/Trehalose – The COGs most associated with the capability to metabolize the sugar trehalose were several maltose-related proteins with correlation scores of 0.94. It has previously been shown that addition of trehalose to growth media induces the maltose system verifying both of these associations [16].

H) G03/Motile – Finally, the pairs related to the G03/Motile phenotype contain proteins involved with chemotaxis and flagella. The result set with a correlation score above 0.8 contained 17 such proteins, and 100% of these were verified by the literature. Similarly, all 5 proteins from the result set with a 0.9 threshold were also confirmed.

Additionally, the 0.8 and 0.9 correlation score threshold data sets for motility were compared with the KEGG database [17,18]. This analysis revealed that 100% of the proteins found to be associated with motility were also annotated as part of the Cell Motility functional classification in the KEGG pathway database.

### Prediction of genes associated to phenotypes

After analyzing the accuracy of the data sets, it is also possible to make reasonable hypotheses for COG-phenotype pairs that are characterized but have not yet been confirmed by the biological literature. These COG-phenotype pairs are listed using their GIDEON identifier/description-COG description/protein name. One example is the B31/Catalase-COG1651/Protein-disulfide isomerase (DsbG) pair with a correlation score of 0.91. Dsb proteins are known to oxidize the sulfhydryl groups of periplasmic proteins to disulfide bonds, donating electrons to ubiquinone, and thereby making the electron transport chain the primary source of oxidizing power for sustaining periplasmic sulfhydryl oxidation [19,20]. During the stationary phase, electron transport to oxygen is reduced. Bandyopadhyay *et al*. suggest a possible complementary role between catalase and the Dsb proteins in maintaining periplasmic sulfhydryl oxidation. It is possible that catalase may be critical in peroxidatically oxidizing ubiquinol or another periplasmic or inner membrane component using H_2_O_2 _as an electron acceptor during the stationary phase when the oxidizing capacity of the electron transport is diminished [21].

With a correlation score of 0.95, other possible associations can be made for the FAP/L-Rhamnose phenotype with various phosphotransferase system sorbitol-specific component proteins. Some microbes such as the Klebsiella I-174 organism make exopolysaccharides with a high rhamnose content [22]. Farres *et al*. showed that the addition of sorbitol increased the production and growth of rhamnose over other carbon sources such as sucrose [23]. This study suggests that proteins involved with sorbitol metabolism and utilization could be linked to rhamnose production.

## Discussion

**Figure 2:**
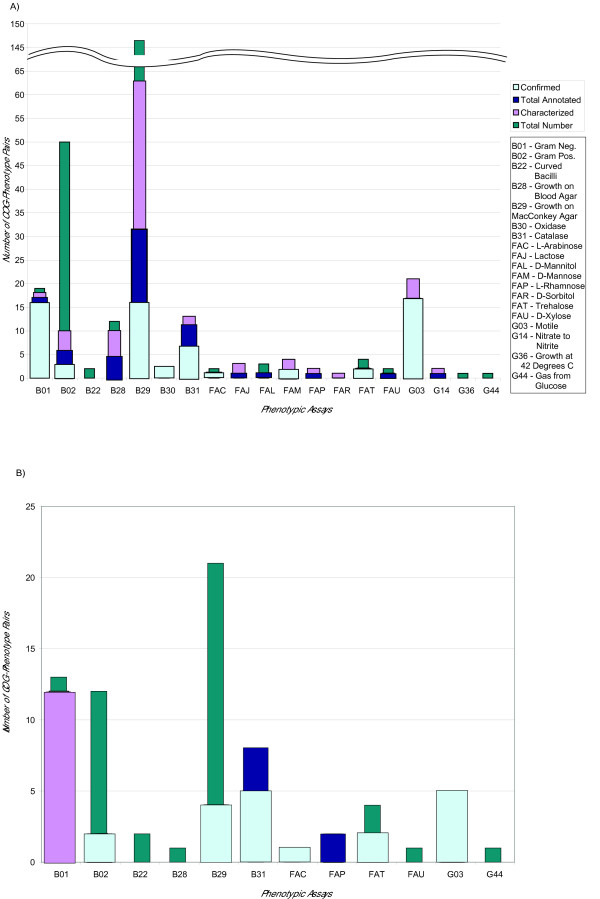
**Number of COG-phenotype associated pairs in each subset of the 0.8 and 0.9 threshold correlation score data sets**. The resulting data sets of the (a) 0.8 correlation threshold and the (b) 0.9 correlation threshold are broken down into four different subsets. Total number (dark blue) is the total number of COG-phenotype associated pairs found at the 0.8 and 0.9 thresholds respectively. Characterized (light purple) refers to those pairs where the COG has a known function. Annotated (blue-green) are those pairs which were selected for literature verification. Finally, confirmed (light blue) are the associations which were validated in the literature. This is shown for each lab indicated by its GIDEON identifier.

Based on the breakdown of total number of associated pairs for each laboratory condition (Figure 2) for the 0.8 correlation data set, the phenotypes that have 10 or more associated COGs have a more likely chance of containing confirmed literature hits. This is roughly 3% of the total number of phenotype-COG pairs. However, there are labs such as B30/Oxidase, FAM/Mannose, and FAT/Trehalose with only 2 results, but all are confirmed at 100%. The 0.9 correlation data set has 86% confirmed associations out of all the characterized pairs, while the 0.8 correlation data set has 66%.

This study reports a percentage of confirmed associations in order to approximate the accuracy of these results. However, this number is most likely a lower bound, since it is possible that some of the predicted associations mentioned in this paper will be experimentally corroborated in the future, raising these percentages.

In addition, although we used the literature as a means of verifying associations, in essence, it is those associations which we were unable to verify that are perhaps the most interesting because these represent new testable hypotheses. By uncovering these novel relationships, it is possible to make inferences about the interrelatedness of what at the outset seem disparate processes. In a similar fashion, for the purpose of assessing our method we were unable to include the COGs with unknown function, but ideally we would like to extend this method to make predictions regarding possible functions of these uncharacterized COGs on the basis of the phenotypes they are most associated with. Finally, while the data in the GIDEON database is extensive, not all assays were performed on all microbes resulting in some missing data.

## Conclusion

This analysis shows that the integration of biological and biomedical information databases can augment and enhance biological understanding. This approach is an introduction to resources that are yet to be fully utilized. Here we describe the combination of a manually annotated phenotype database, GIDEON, with the well-documented COGs database to find new associations between a certain phenotype and an organism's genotype. This study reports phenotype-COG associations found above a certain threshold. We have demonstrated that the method is able to detect known phenotype-COG relationships, as well as, discover new ones. Additional comparisons with the KEGG database confirm the resulting COGs shown to be related to the motility phenotype, and subsequently, further predictions are made for potential phenotype-COGs pairs that have not yet been discovered.

These results suggest a new direction for inferring either the phenotype or genotype of an uncharacterized organism. This approach can further be applied to discovering relationships between the pathogenicity of these organisms to functionally related proteins. Moreover, this type of analysis could be extended beyond phenotype-genotype to phenotype-drug design by associating molecules to their phenotypic effects. By integrating clinical and biological databases, additional studies can be developed to further the understanding of phenotypic relationships and, in turn, augment the medical community's ability to rapidly identify infectious agents.

## Methods

### Mapping organisms between databases

The laboratory results in the GIDEON database are primarily used for identifying bacterial species for medical diagnostics. Since different strains of the same bacterial species are often sequenced, NCBI's taxonomic annotation is sometimes at the subspecies level. In contrast, the GIDEON phenotypes do not achieve such a high resolution, and for this reason GIDEON taxonomic annotation is established at the level where the phenotype is consistent in all descendants of the phylogenetic tree (generally the species level).

This presented a complication in integrating the two data sources. To overcome this, we assumed that phenotypes from the microbiological database for one species are valid for every subsumed subspecies and strain listed in the COGs database. This is a valid assumption since the GIDEON dataset provides microbiologists with relevant tests designed to distinguish between organisms according to their phenotypes. Thus if the phenotype is specific to a subspecies, it will be annotated at the level of the subspecies, but if the phenotype is common to all subspecies, it is recorded at the level of the species.

Following this principle, we first identified the taxonomic level for the fully sequenced bacteria in the COGs dataset, and then used text string matching followed by manual examination to map the species in GIDEON [24]. As a result, we have mapped the 37 microorganisms present in both GIDEON and the COGs. Of the 37 mappings, 23 have identical species annotations in GIDEON and COGs, and 9 have a species annotation in GIDEON mapped to one or more subspecies in COGs.

There were several COGs species including *H. pylori*, *E. coli*, *M. tuberculosis*, and *N. meningitidis *which had more than one subspecies with complete genome sequences. In these cases, the subspecies were merged to the single GIDEON species by selecting only the COGs common to all subspecies. In this manner, we eliminated the subspecies specific differences that the phenotypic assays would have been unable to resolve. We generated a matrix showing the presence and absence of COGs across these 37 species.

### Associating genes to phenotypes

We employed a correlation analysis to quantify the association between a given COG and a GIDEON phenotype. Two matrices were constructed. We defined *X *as a two-dimensional matrix indicating the presence or absence of organisms within a COG (*X *was constructed as an M × N matrix, where M is equal to the number of COGs and N is equal to the number of organisms within a COG). For the corresponding GIDEON lab conditions, a similar distance matrix, Y, was constructed as an N × L matrix, where N is equal to the percent survival of organisms subjected to each lab condition and L is equal to the number of lab conditions. *X*_*ij *_is the presence or absence of a COG *m*_*i *_within an organism *n*_*j*_, and *Y*_*jk *_signifies the percent response of an organism *n*_*j *_under a certain lab condition *l*_*k*_. We computed an M × L matrix of linear correlation coefficients *r*_*ik *_(Pearson's correlation coefficient [25], where each *r*_*ik *_is defined as:

rik=∑j=1N(Xij−X¯i)(Yjk−Y¯k)∑j=1N(Xij−X¯i)2∑j=1N(Yjk−Y¯k)2
 MathType@MTEF@5@5@+=feaafiart1ev1aaatCvAUfKttLearuWrP9MDH5MBPbIqV92AaeXatLxBI9gBaebbnrfifHhDYfgasaacH8akY=wiFfYdH8Gipec8Eeeu0xXdbba9frFj0=OqFfea0dXdd9vqai=hGuQ8kuc9pgc9s8qqaq=dirpe0xb9q8qiLsFr0=vr0=vr0dc8meaabaqaciaacaGaaeqabaqabeGadaaakeaacqWGYbGCdaWgaaWcbaGaemyAaKMaem4AaSgabeaakiabg2da9maalaaabaWaaabCaeaadaqadaqaaiabdIfaynaaBaaaleaacqWGPbqAcqWGQbGAaeqaaOGaeyOeI0IafmiwaGLbaebadaWgaaWcbaGaemyAaKgabeaaaOGaayjkaiaawMcaaaWcbaGaemOAaOMaeyypa0JaeGymaedabaGaemOta4eaniabggHiLdGcdaqadaqaaiabdMfaznaaBaaaleaacqWGQbGAcqWGRbWAaeqaaOGaeyOeI0IafmywaKLbaebadaWgaaWcbaGaem4AaSgabeaaaOGaayjkaiaawMcaaaqaamaakaaabaWaaabCaeaadaqadaqaaiabdIfaynaaBaaaleaacqWGPbqAcqWGQbGAaeqaaOGaeyOeI0IafmiwaGLbaebadaWgaaWcbaGaemyAaKgabeaaaOGaayjkaiaawMcaamaaCaaaleqabaGaeGOmaidaaaqaaiabdQgaQjabg2da9iabigdaXaqaaiabd6eaobqdcqGHris5aaWcbeaakmaakaaabaWaaabCaeaadaqadaqaaiabdMfaznaaBaaaleaacqWGQbGAcqWGRbWAaeqaaOGaeyOeI0IafmywaKLbaebadaWgaaWcbaGaem4AaSgabeaaaOGaayjkaiaawMcaamaaCaaaleqabaGaeGOmaidaaaqaaiabdQgaQjabg2da9iabigdaXaqaaiabd6eaobqdcqGHris5aaWcbeaaaaaaaa@6ED3@

with -1≤*r*_*ik*_≤+1 where X¯
 MathType@MTEF@5@5@+=feaafiart1ev1aaatCvAUfKttLearuWrP9MDH5MBPbIqV92AaeXatLxBI9gBaebbnrfifHhDYfgasaacH8akY=wiFfYdH8Gipec8Eeeu0xXdbba9frFj0=OqFfea0dXdd9vqai=hGuQ8kuc9pgc9s8qqaq=dirpe0xb9q8qiLsFr0=vr0=vr0dc8meaabaqaciaacaGaaeqabaqabeGadaaakeaacuWGybawgaqeaaaa@2DFD@_i _is the mean over all *X*_*ij*_'s for all j from 1..N, and Y¯
 MathType@MTEF@5@5@+=feaafiart1ev1aaatCvAUfKttLearuWrP9MDH5MBPbIqV92AaeXatLxBI9gBaebbnrfifHhDYfgasaacH8akY=wiFfYdH8Gipec8Eeeu0xXdbba9frFj0=OqFfea0dXdd9vqai=hGuQ8kuc9pgc9s8qqaq=dirpe0xb9q8qiLsFr0=vr0=vr0dc8meaabaqaciaacaGaaeqabaqabeGadaaakeaacuWGzbqwgaqeaaaa@2DFF@_k _is the mean over all *Y*_*jk*_'s all j from 1..N. In our context, *X*_*ij *_and *Y*_*jk *_are presence or absence of a COG within an organism or the percent response of an organism subject to adverse growth condition, respectively.

While the correlation measures the strength of association between an organism's genomic content and its phenotype, we also applied another method, exploiting the hypergeometric distribution function, to determine the significance of these associations

p(i≥m|N,M,n)=∑i=mn(Mi)(M−nn−i)(Nn)
 MathType@MTEF@5@5@+=feaafiart1ev1aaatCvAUfKttLearuWrP9MDH5MBPbIqV92AaeXatLxBI9gBaebbnrfifHhDYfgasaacH8akY=wiFfYdH8Gipec8Eeeu0xXdbba9frFj0=OqFfea0dXdd9vqai=hGuQ8kuc9pgc9s8qqaq=dirpe0xb9q8qiLsFr0=vr0=vr0dc8meaabaqaciaacaGaaeqabaqabeGadaaakeaacqWGWbaCcqGGOaakcqWGPbqAcqGHLjYScqWGTbqBcqGG8baFcqWGobGtcqGGSaalcqWGnbqtcqGGSaalcqWGUbGBcqGGPaqkcqGH9aqpdaaeWbqaamaalaaabaWaaeWaaqaabeqaaiabd2eanbqaaiabdMgaPbaacaGLOaGaayzkaaWaaeWaaqaabeqaaiabd2eanjabgkHiTiabd6gaUbqaaiabd6gaUjabgkHiTiabdMgaPbaacaGLOaGaayzkaaaabaWaaeWaaqaabeqaaiabd6eaobqaaiabd6gaUbaacaGLOaGaayzkaaaaaaWcbaGaemyAaKMaeyypa0JaemyBa0gabaGaemOBa4ganiabggHiLdaaaa@5493@

where N is the total number of species, n is the number of species that are positive in the laboratory result, M is the number of species that have the COGs family, and m is the number of species that have the COGs family and is also positive in the laboratory result, where a result ≤ 20% response is considered negative. So for a given gene found in M species, the hypergeometric function provides the probability by random chance that the gene is found in *m *species which contain the COG and are also positive in the laboratory test.

### Assessment of predicted results

The following criteria were applied to the correlated data set. The intersection between a specific COG and a phenotype had to contain at least 3 organisms, and for any intersection, 30% of the microbes had to share the COG. The scores were adjusted using the standard Bonferroni error correction for multiple testing. Since the Bonferroni correction is one of the most conservative, it is likely that some biologically relevant associations were unnecessarily discarded. In this case α was set as less than equal to 0.01, therefore, any hypergeometric distribution score less than or equal to 0.0001 was deemed significant. Using these criteria, we set a 0.8 and a 0.9 correlation threshold to assess the significance of the COG-phenotype associations.

For the 0.8 correlation threshold, 290 total associations were obtained. We identified a subset of these data (154) that contain only COGs that have a known function. Of these 154 pairs, we performed detailed literature searches on 100 randomly selected pairs to confirm the validity of the positive COG-phenotype associations.

There were 74 associations found in the 0.9 correlation data set. Thirty-six of these pairs contained a COG of known function. Literature searches were performed on all 36 associations.

### Website

All the GIDEON data used in this analysis is freely available from  .

## List of Abbreviations

Clusters of Orthologous Groups (COGs), National Center for Biotechnology Information (NCBI)

## Supplementary Material

Additional file 1 Organism mapping between GIDEON and COGs species analyzed. Click here for file

Additional file 2 Correlation and hypergeometric distribution scores for complete data set with correlation above 0.8. Click here for file

Additional file 3 Correlation and hypergeometric distribution scores for annotated data set with correlation above 0.8. Click here for file

Additional file 4 GIDEON laboratory tests analyzed and their descriptions. Click here for file

Additional file 5 COGs analyzed and their descriptions. Click here for file

## Figures and Tables

**Figure 1:**
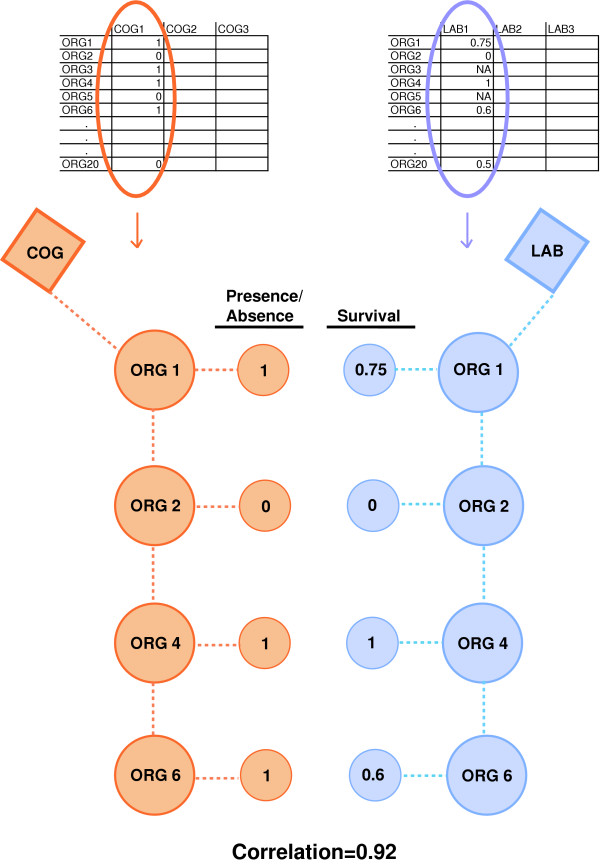
**Diagram of correlation analysis for associating COGs to lab condition phenotypes**. The correlation analysis measures the association between a COG's organism profile (presence or absence of an organism) and a lab condition's organism survival profile. Organisms that have a COG (red) are mapped to the organism's response to adverse growth conditions (blue) creating two vectors that are used for the correlation calculation.

**Table 1:** Number of validated associations at the 0.8 and 0.9 threshold

**Data Set**	**Total Number of Associated Pairs**	**Number of COGs with Known Function**	**Number of Pairs Randomly Selected for Literature Search**	**Number of Pairs with Confirmed Association in the Literature**	**% Confirmed Pairs**
Corr Scores ≥0.8	290	154	100	66	66%
Corr Scores ≥0.9	74	36	36	31	86%

**Table 2:** Accuracy of associations confirmed by literature broken down by individual condition. Characterized are those pairs where the COG has a known function. Confirmed are those associations that were verified in the literature.

**Lab/Condition**	**Correlation Above 0.8**			**Correlation Above 0.9**		
	
	**Total Characterized Pairs**	**Confirmed Pair Associations**	**Percent Confirmed**	**Total Characterized Pairs**	**Confirmed Pair Associations**	**Percent Confirmed**
**B01/Gram-negative**	17	16	94%	12	12	100%
**B02/Gram-positive**	6	3	50%	2	2	100%
**B28/Growth on Ordinary Blood Agar**	5	0	0%	NA	NA	NA
**B29/Growth on MacConkey Agar**	31	16	52%	4	4	100%
**B30/Oxidase**	2	2	100%	NA	NA	NA
**B31/Catalase**	11	7	64%	8	5	63%
**FAC/L-Arabinose**	1	1	100%	1	1	100%
**FAJ/Lactose**	1	0	0%	NA	NA	NA
**FAL/D-Mannitol**	1	0	0%	NA	NA	NA
**FAM/D-Mannose**	2	2	100%	NA	NA	NA
**FAP/L-Rhamnose**	1	0	0%	2	0	0%
**FAT/Trehalose**	2	2	100%	2	2	100%
**FAU/D-Xylose**	1	0	0%	NA	NA	NA
**G03/Motile**	17	17	100%	5	5	100%
**G14/Nitrate to Nitrite**	1	0	0%	NA	NA	NA

**Table 3:** Overview of representative hits above 0.8

**Lab/Condition**	**Cog/Protein Name**	**Correlation**	**P-value**	**Protein Function**
**B01/Gram-negative**	COG0763/Lipid A disaccharide synthetase	0.95	1.71E-09	Involved in Lipid A biosynthesis [26–28]
	COG0774/UDP-3-O-acyl-N-acetylglucosamine deacetylase	0.95	1.71E-09	Involved in Lipid A biosynthesis [29] [27,30]
	COG1212/CMP-2-keto-3-deoxyoctulosonic acid synthetase	0.95	1.71E-09	Involved in lipopolysaccharide biosynthesis [31]
	COG2877/3-deoxy-D-manno-octulosonic acid (KDO) 8-phosphate synthase	0.95	1.71E-09	Involved in lipopolysaccharide biosynthesis [32]
	COG0848/Biopolymer transport protein	0.95	1.71E-09	Outer membrane transporters [33,34]
	COG2885/Outer membrane protein	0.84	2.46E-09	Outer membrane protein [35]
**B02/Gram-positive**	COG3764/Sortase	1.0	2.59E-08	Plasma membrane protein [36]
	COG2344/AT-rich DNA-binding protein	0.92	7.77E-07	Transcriptional regulator [37]
	COG3966/Protein involved in D-alanine esterification of lipoteichoic acid and wall teichoic acid (D-alanine transfer protein)	0.84	1.2E-05	Cell wall and membrane component protein [38]
**B29/Growth on MacConkey Agar**	COG4206/Outer membrane cobalamin receptor protein	0.99	8.04E-09	Outer membrane protein [39]
	COG4787/Flagellar basal body rod protein	0.97	2.33E-07	Periplasmic protein [40]
	COG3166/Tfp pilus assembly protein PilN	0.83	9.77E-06	Outer membrane proteins [41]
**B30/Oxidase**	COG3278/Cbb3-type cytochrome oxidase, subunit 1	0.85	7.84E-06	Oxidase protein subunit
**B30/Oxidase**	COG2993/Cbb3-type cytochrome oxidase, cytochrome c subunit	0.85	7.84E-06	Oxidase protein subunit
**B31/Catalase**	COG0753/Catalase	0.97	7.69E-06	Peroxisomal Marker Enzyme
	COG1607/Acyl-CoA hydrolase	0.97	7.69E-06	Enzyme involved in lipid metabolism [13]
**FAC/L-Arabinose**	COG3717/5-keto 4-deoxyuronate isomerase	0.97	3.95E-05	Enzyme involved in carbohydrate metabolism [14,15]
**FAM/D-Mannose**	COG0246/Mannitol-1-phosphate/altronate dehydrogenases	0.85	4.68E-05	Oxidizes mannitol to mannose [42]
**FAT/Trehalose**	COG2182/Maltose-binding periplasmic proteins/domains	0.94	3.4E-05	Maltose-related protein [16]
**G03/Motile**	COG0835/Chemotaxis signal transduction protein	0.94	4.93E-09	Chemotaxis-related protein
	COG1345/Flagellar capping protein	0.94	4.93E-09	Flagella-related protein
	COG1516/Flagellin-specific chaperone FliS	0.94	4.93E-09	Flagella-related protein
