# Supplementary material for: Integration of curated databases to identify genotype-phenotype associations
Source: BMC Genomics. 2006 Oct 12;7:257. doi: 10.1186/1471-2164-7-257 (PMC1630430; doi:10.1186/1471-2164-7-257)
Supplement: Additional file 1 — Organism mapping between GIDEON and COGs species analyzed. [file 1471-2164-7-257-S1.pdf]

Additional Table 1. Mapping COG species to GIDEON species

| Gideon Species | COGs Species | NCBI Taxonomy ID |
|---|---|---|
| Actinobacteria Corynebacterium glutamicum | Actinobacteria Corynebacterium glutamicum | 1718 |
| Actinobacteria Mycobacterium leprae | Actinobacteria Mycobacterium leprae | 1769 |
| alpha Brucella melitensis | alpha Brucella melitensis | 29459 |
| alpha Rickettsia conorii | alpha Rickettsia conorii | 781 |
| alpha Rickettsia prowazekii | alpha Rickettsia prowazekii | 782 |
| Bacteria Fusobacterium nucleatum | Bacteria Fusobacterium nucleatum | 190304 |
| Chlam-Spir Borrelia burgdorferi | Chlam-Spir Borrelia burgdorferi | 139 |
| Chlam-Spir Chlamydia trachomatis | Chlam-Spir Chlamydia trachomatis | 813 |
| Chlam-Spir Chlamydophila pneumoniae | Chlam-Spir Chlamydophila pneumoniae CWL029 | 115713 |
| Chlam-Spir Treponema pallidum | Chlam-Spir Treponema pallidum | 160 |
| gamma Haemophilus influenzae | gamma Haemophilus influenzae | 71421 |
| gamma Pasteurella multocida | gamma Pasteurella multocida | 747 |
| gamma Pseudomonas aeruginosa | gamma Pseudomonas aeruginosa | 287 |
| gamma Salmonella typhimurium | gamma Salmonella typhimurium LT2 | 99287 |
| gamma Vibrio cholerae | gamma Vibrio cholerae | 666 |
| gamma Yersinia pestis | gamma Yersinia pestis | 632 |
| Gramplus Bacillus subtilis | Gramplus Bacillus subtilis | 1423 |
| Gramplus Clostridium acetobutylicum | Gramplus Clostridium acetobutylicum | 1488 |
| Gramplus Lactococcus lactis | Gramplus Lactococcus lactis | 1360 |
| Gramplus Listeria innocua | Gramplus Listeria innocua | 1642 |
| Gramplus Mycoplasma genitalium | Gramplus Mycoplasma genitalium | 2097 |
| Gramplus Mycoplasma pneumoniae | Gramplus Mycoplasma pneumoniae | 2104 |
| Gramplus Staphylococcus aureus | Gramplus Staphylococcus aureus N315 | 158879 |
| Gramplus Streptococcus pneumoniae | Gramplus Streptococcus pneumoniae TIGR4 | 170187 |
| Gramplus Streptococcus pyogenes | Gramplus Streptococcus pyogenes M1 GAS | 160490 |
| Gramplus Ureaplasma urealyticum | Gramplus Ureaplasma urealyticum | 2130 |
| Proteobacteria Campylobacter jejuni | Proteobacteria Campylobacter jejuni | 197 |
| Proteobacteria Ralstonia solanacearum | Proteobacteria Ralstonia solanacearum | 305 |

| Gideon Species | COGs Subspecies | NCBI Taxonomy ID |
|---|---|---|
| Actinobacteria Mycobacterium tuberculosis | Actinobacteria Mycobacterium tuberculosis CDC1551 | 83331 |
| | Actinobacteria Mycobacterium tuberculosis H37Rv | 83332 |
| gamma Escherichia coli | gamma Escherichia coli K12 | 83333 |
| | gamma Escherichia coli O157:H7 | 83334 |
| | gamma Escherichia coli O157:H7 EDL933 | 155864 |
| Proteobacteria Helicobacter pylori | Proteobacteria Helicobacter pylori 26695 | 85962 |
| | Proteobacteria Helicobacter pylori J99 | 85963 |
| Proteobacteria Neisseria meningitidis | Proteobacteria Neisseria meningitidis Z2491 | 122587 |
| | Proteobacteria Neisseria meningitidis MC58 | 122586 |
