# Supplementary material for: Integration of curated databases to identify genotype-phenotype associations
Source: BMC Genomics. 2006 Oct 12;7:257. doi: 10.1186/1471-2164-7-257 (PMC1630430; doi:10.1186/1471-2164-7-257)
Supplement: Additional file 2 — Correlation and hypergeometric distribution scores for complete data set with correlation above 0.8. [file 1471-2164-7-257-S2.pdf]

Additional Table 2. Correlation and Hypergeometric Distribution Scores for Complete Data Set with Correlation above 0.8

COG	Gideon Lab	Correlation	Hypergeometric
COG0763	B01	0.945234	1.71E-09
COG0774	B01	0.945234	1.71E-09
COG0811	B01	0.945234	1.71E-09
COG0848	B01	0.945234	1.71E-09
COG1043	B01	0.945234	1.71E-09
COG1044	B01	0.945234	1.71E-09
COG1212	B01	0.945234	1.71E-09
COG1519	B01	0.945234	1.71E-09
COG1663	B01	0.945234	1.71E-09
COG2877	B01	0.945234	1.71E-09
COG4775	B01	0.944614	2.46E-09
COG0795	B01	0.944614	2.46E-09
COG1137	B01	0.944614	2.46E-09
COG2204	B01	0.887139	3.52E-08
COG1538	B01	0.846623	8.71E-08
COG2885	B01	0.838831	2.46E-07
COG1495	B01	0.83364	2.69E-07
COG0823	B01	0.801482	4.14E-07
COG4105	B01	0.801482	4.14E-07
COG3764	B02	0.997591	2.59E-08
COG3887	B02	0.917477	7.77E-07
COG1098	B02	0.917477	7.77E-07
COG3857	B02	0.917477	7.77E-07
COG3935	B02	0.917477	7.77E-07
COG4109	B02	0.917477	7.77E-07
COG4463	B02	0.917477	7.77E-07
COG4465	B02	0.917477	7.77E-07
COG2344	B02	0.917477	7.77E-07
COG3601	B02	0.917477	7.77E-07
COG3906	B02	0.917477	7.77E-07
COG4466	B02	0.917477	7.77E-07
COG3599	B02	0.843121	4.27E-06
COG1511	B02	0.843121	4.27E-06
COG1358	B02	0.836268	6.01E-06
COG1476	B02	0.836268	6.01E-06
COG1323	B02	0.836268	6.01E-06
COG1939	B02	0.836268	6.01E-06
COG3481	B02	0.836268	6.01E-06
COG4224	B02	0.836268	6.01E-06
COG3966	B02	0.835606	1.20E-05
COG4473	B02	0.835606	1.20E-05
COG4477	B02	0.835606	1.20E-05
COG4537	B02	0.835606	1.20E-05
COG4839	B02	0.835606	1.20E-05
COG4862	B02	0.835606	1.20E-05

COG5002	B02	0.835606	1.20E-05
COG4098	B02	0.835606	1.20E-05
COG1668	B02	0.835606	1.20E-05
COG3853	B02	0.835606	1.20E-05
COG2179	B02	0.835606	1.20E-05
COG2339	B02	0.835606	1.20E-05
COG3679	B02	0.835606	1.20E-05
COG3883	B02	0.835606	1.20E-05
COG4116	B02	0.835606	1.20E-05
COG4199	B02	0.835606	1.20E-05
COG4467	B02	0.835606	1.20E-05
COG4469	B02	0.835606	1.20E-05
COG4470	B02	0.835606	1.20E-05
COG4471	B02	0.835606	1.20E-05
COG4472	B02	0.835606	1.20E-05
COG4474	B02	0.835606	1.20E-05
COG4476	B02	0.835606	1.20E-05
COG4479	B02	0.835606	1.20E-05
COG4483	B02	0.835606	1.20E-05
COG4758	B02	0.835606	1.20E-05
COG4768	B02	0.835606	1.20E-05
COG4856	B02	0.835606	1.20E-05
COG5503	B02	0.835606	1.20E-05
COG2357	B02	0.828591	4.27E-06
COG3014	B22	1	1.51E-05
COG3018	B22	1	1.51E-05
COG1188	B28	0.94018	7.02E-09
COG0826	B28	0.880267	8.76E-08
COG2264	B28	0.880267	8.76E-08
COG0673	B28	0.842956	1.57E-07
COG0458	B28	0.836567	8.21E-07
COG0505	B28	0.836567	8.21E-07
COG0818	B28	0.825486	5.88E-07
COG1135	B28	0.823724	8.21E-07
COG1464	B28	0.823724	8.21E-07
COG2011	B28	0.823724	8.21E-07
COG2252	B28	0.823724	8.21E-07
COG1576	B28	0.818336	1.01E-06
COG3852	B29	0.989446	8.04E-09
COG2967	B29	0.989446	8.04E-09
COG3127	B29	0.989446	8.04E-09
COG3154	B29	0.989446	8.04E-09
COG4206	B29	0.989446	8.04E-09
COG4651	B29	0.989446	8.04E-09
COG3242	B29	0.989446	8.04E-09
COG3313	B29	0.989446	8.04E-09
COG4783	B29	0.989446	8.04E-09
COG3143	B29	0.970157	2.33E-07
COG4787	B29	0.970157	2.33E-07

COG4181	B29	0.970157	2.33E-07
COG3123	B29	0.970157	2.33E-07
COG3147	B29	0.970157	2.33E-07
COG3157	B29	0.970157	2.33E-07
COG3164	B29	0.970157	2.33E-07
COG4172	B29	0.921125	8.04E-08
COG3895	B29	0.911335	2.33E-07
COG3605	B29	0.909745	2.33E-07
COG3311	B29	0.909745	2.33E-07
COG3126	B29	0.909745	2.33E-07
COG3150	B29	0.897923	2.03E-06
COG3159	B29	0.897923	2.03E-06
COG3678	B29	0.894872	2.03E-06
COG3951	B29	0.894872	2.03E-06
COG2105	B29	0.894872	2.03E-06
COG5006	B29	0.894872	2.03E-06
COG4579	B29	0.893708	3.50E-06
COG3228	B29	0.893708	3.50E-06
COG4681	B29	0.893708	3.50E-06
COG5393	B29	0.893708	3.50E-06
COG5544	B29	0.893708	3.50E-06
COG2943	B29	0.892037	3.50E-06
COG3418	B29	0.892037	3.50E-06
COG0417	B29	0.892037	3.50E-06
COG3130	B29	0.892037	3.50E-06
COG3160	B29	0.892037	3.50E-06
COG4568	B29	0.892037	3.50E-06
COG2716	B29	0.892037	3.50E-06
COG2988	B29	0.892037	3.50E-06
COG3131	B29	0.892037	3.50E-06
COG3138	B29	0.892037	3.50E-06
COG3155	B29	0.892037	3.50E-06
COG4580	B29	0.892037	3.50E-06
COG4592	B29	0.892037	3.50E-06
COG4598	B29	0.892037	3.50E-06
COG3124	B29	0.892037	3.50E-06
COG3129	B29	0.892037	3.50E-06
COG3132	B29	0.892037	3.50E-06
COG3136	B29	0.892037	3.50E-06
COG3139	B29	0.892037	3.50E-06
COG3141	B29	0.892037	3.50E-06
COG3148	B29	0.892037	3.50E-06
COG3151	B29	0.892037	3.50E-06
COG3217	B29	0.892037	3.50E-06
COG3266	B29	0.892037	3.50E-06
COG3272	B29	0.892037	3.50E-06
COG3455	B29	0.892037	3.50E-06
COG3501	B29	0.892037	3.50E-06
COG3515	B29	0.892037	3.50E-06

COG3516	B29	0.892037	3.50E-06
COG3517	B29	0.892037	3.50E-06
COG3518	B29	0.892037	3.50E-06
COG3519	B29	0.892037	3.50E-06
COG3520	B29	0.892037	3.50E-06
COG3521	B29	0.892037	3.50E-06
COG3522	B29	0.892037	3.50E-06
COG3523	B29	0.892037	3.50E-06
COG3776	B29	0.892037	3.50E-06
COG4582	B29	0.892037	3.50E-06
COG0408	B29	0.861205	4.42E-07
COG2391	B29	0.861205	4.42E-07
COG2960	B29	0.861205	4.42E-07
COG3045	B29	0.861205	4.42E-07
COG4174	B29	0.861205	4.42E-07
COG3206	B29	0.839958	2.03E-06
COG1953	B29	0.838432	2.03E-06
COG3026	B29	0.832705	9.77E-06
COG3073	B29	0.832705	9.77E-06
COG3116	B29	0.832705	9.77E-06
COG4795	B29	0.832705	9.77E-06
COG2925	B29	0.832705	9.77E-06
COG3025	B29	0.832705	9.77E-06
COG4117	B29	0.83021	3.50E-06
COG5281	B29	0.83021	3.50E-06
COG2747	B29	0.829758	9.77E-06
COG3144	B29	0.829758	9.77E-06
COG3166	B29	0.829758	9.77E-06
COG4967	B29	0.829758	9.77E-06
COG0429	B29	0.829758	9.77E-06
COG3009	B29	0.829758	9.77E-06
COG4147	B29	0.829758	9.77E-06
COG4150	B29	0.828539	3.50E-06
COG2109	B29	0.821864	1.77E-06
COG4575	B29	0.815948	2.61E-05
COG3726	B29	0.814358	2.61E-05
COG3539	B29	0.813958	3.61E-05
COG3725	B29	0.813958	3.61E-05
COG4650	B29	0.813958	3.61E-05
COG4790	B29	0.813958	3.61E-05
COG5404	B29	0.813958	3.61E-05
COG0430	B29	0.813958	3.61E-05
COG3724	B29	0.813958	3.61E-05
COG4573	B29	0.813958	3.61E-05
COG3122	B29	0.813958	3.61E-05
COG3134	B29	0.813958	3.61E-05
COG3497	B29	0.813958	3.61E-05
COG3500	B29	0.813958	3.61E-05
COG3784	B29	0.813958	3.61E-05

COG3960	B29	0.813958	3.61E-05
COG4269	B29	0.813958	3.61E-05
COG4672	B29	0.813958	3.61E-05
COG4718	B29	0.813958	3.61E-05
COG4723	B29	0.813958	3.61E-05
COG4733	B29	0.813958	3.61E-05
COG5435	B29	0.813958	3.61E-05
COG3121	B29	0.812768	2.61E-05
COG3188	B29	0.812768	2.61E-05
COG3137	B29	0.812182	3.61E-05
COG3248	B29	0.812182	3.61E-05
COG4238	B29	0.812182	3.61E-05
COG4999	B29	0.812182	3.61E-05
COG1446	B29	0.812182	3.61E-05
COG3954	B29	0.812182	3.61E-05
COG4138	B29	0.812182	3.61E-05
COG4139	B29	0.812182	3.61E-05
COG1986	B29	0.812182	3.61E-05
COG2425	B29	0.812182	3.61E-05
COG3530	B29	0.812182	3.61E-05
COG3788	B29	0.812182	3.61E-05
COG3604	B29	0.811178	2.61E-05
COG2356	B29	0.811178	2.61E-05
COG2515	B29	0.811178	2.61E-05
COG4771	B29	0.811178	2.61E-05
COG4779	B29	0.811178	2.61E-05
COG0397	B29	0.811178	2.61E-05
COG3047	B29	0.810679	1.77E-06
COG3038	B29	0.810679	1.77E-06
COG1261	B29	0.807883	1.77E-06
COG2063	B29	0.807883	1.77E-06
COG2360	B29	0.807883	1.77E-06
COG1838	B29	0.807883	1.77E-06
COG1951	B29	0.807883	1.77E-06
COG2066	B29	0.807883	1.77E-06
COG3158	B29	0.807883	1.77E-06
COG4239	B29	0.807883	1.77E-06
COG3278	B30	0.851245	7.84E-06
COG2993	B30	0.851245	7.84E-06
COG0753	B31	0.967324	7.69E-06
COG1607	B31	0.967324	7.69E-06
COG0805	B31	0.906479	1.71E-07
COG1651	B31	0.906479	1.71E-07
COG1826	B31	0.906479	1.71E-07
COG0328	B31	0.906479	1.71E-07
COG1825	B31	0.906479	1.71E-07
COG0114	B31	0.906479	1.71E-07
COG0861	B31	0.829295	3.93E-06
COG0407	B31	0.828355	1.54E-06

COG0755	B31	0.820158	1.54E-06
COG0479	B31	0.820158	1.54E-06
COG0746	B31	0.820158	1.54E-06
COG3717	FAC	0.970445	3.95E-05
COG2850	FAC	0.800014	7.49E-05
COG3684	FAJ	0.838533	9.22E-05
COG3723	FAJ	0.810417	9.22E-05
COG4570	FAJ	0.810417	9.22E-05
COG1801	FAL	0.827708	4.92E-05
COG3726	FAL	0.827142	5.31E-05
COG2356	FAL	0.818329	5.31E-05
COG0246	FAM	0.849961	4.69E-05
COG1175	FAM	0.849961	4.69E-05
COG1263	FAM	0.849961	4.69E-05
COG1264	FAM	0.849961	4.69E-05
COG3730	FAP	0.953977	2.58E-05
COG3731	FAP	0.953977	2.58E-05
COG3717	FAR	0.871545	6.93E-05
COG2182	FAT	0.938041	3.40E-05
COG3833	FAT	0.938041	3.40E-05
COG1956	FAT	0.902745	4.42E-05
COG3091	FAT	0.902745	4.42E-05
COG2850	FAU	0.945358	3.37E-06
COG3133	FAU	0.8475	2.69E-05
COG0643	G03	0.935693	4.93E-09
COG0835	G03	0.935693	4.93E-09
COG0840	G03	0.935693	4.93E-09
COG1345	G03	0.935693	4.93E-09
COG1516	G03	0.935693	4.93E-09
COG1256	G03	0.882266	4.19E-08
COG1291	G03	0.882266	4.19E-08
COG1344	G03	0.882266	4.19E-08
COG1360	G03	0.882266	4.19E-08
COG1558	G03	0.882266	4.19E-08
COG1677	G03	0.882266	4.19E-08
COG1684	G03	0.882266	4.19E-08
COG1766	G03	0.882266	4.19E-08
COG1815	G03	0.882266	4.19E-08
COG1843	G03	0.882266	4.19E-08
COG1868	G03	0.882266	4.19E-08
COG1987	G03	0.882266	4.19E-08
COG4786	G03	0.882266	4.19E-08
COG1580	G03	0.870082	9.24E-08
COG1749	G03	0.83331	7.02E-07
COG1352	G03	0.806357	9.05E-07
COG2166	G14	0.851828	3.37E-05
COG1169	G14	0.827001	6.12E-05
COG0679	G36	0.878941	8.08E-05
COG2327	G44	0.99665	4.91E-05
