# Supplementary material for: Integration of curated databases to identify genotype-phenotype associations
Source: BMC Genomics. 2006 Oct 12;7:257. doi: 10.1186/1471-2164-7-257 (PMC1630430; doi:10.1186/1471-2164-7-257)
Supplement: Additional file 3 — Correlation and hypergeometric distribution scores for annotated data set with correlation above 0.8. [file 1471-2164-7-257-S3.pdf]

Additional Table 3. Correlation and Hypergeometric Distribution Scores for Annotated Data Set with Correlation above 0.8

COG	Gideon Lab	Correlation	Hypergeometric
COG2877	B01	0.95	1.71E-09
COG1519	B01	0.95	1.71E-09
COG0848	B01	0.95	1.71E-09
COG0763	B01	0.95	1.71E-09
COG2877	B01	0.95	1.71E-09
COG1044	B01	0.95	1.71E-09
COG1043	B01	0.95	1.71E-09
COG0848	B01	0.95	1.71E-09
COG1663	B01	0.95	1.71E-09
COG1212	B01	0.95	1.71E-09
COG0811	B01	0.95	1.71E-09
COG0774	B01	0.95	1.71E-09
COG4775	B01	0.94	2.46E-09
COG2204	B01	0.89	3.52E-08
COG1538	B01	0.85	8.71E-08
COG1495	B01	0.83	2.69E-07
COG0823	B01	0.80	4.14E-07
COG3764	B02	1.00	2.59E-08
COG2344	B02	0.92	7.77E-07
COG3599	B02	0.84	4.27E-06
COG1358	B02	0.84	6.01E-06
COG3966	B02	0.84	1.20E-05
COG1668	B02	0.84	1.20E-05
COG1188	B28	0.94	7.02E-09
COG2264	B28	0.88	8.76E-08
COG0458	B28	0.84	8.21E-07
COG0818	B28	0.83	5.88E-07
COG1464	B28	0.82	8.21E-07
COG3143	B29	0.97	2.33E-07
COG4206	B29	0.99	8.04E-09
COG2988	B29	0.89	3.50E-06
COG0417	B29	0.89	3.50E-06
COG3418	B29	0.89	3.50E-06
COG3131	B29	0.89	3.50E-06
COG3130	B29	0.89	3.50E-06
COG3160	B29	0.89	3.50E-06
COG0408	B29	0.86	4.42E-07
COG1953	B29	0.84	2.03E-06
COG3073	B29	0.83	9.77E-06
COG3026	B29	0.83	9.77E-06
COG2747	B29	0.83	9.77E-06
COG3166	B29	0.83	9.77E-06
COG2109	B29	0.82	1.77E-06
COG3539	B29	0.81	3.61E-05
COG0430	B29	0.81	3.61E-05

COG3121	B29	0.81	2.61E-05
COG3137	B29	0.81	3.61E-05
COG3248	B29	0.81	3.61E-05
COG4238	B29	0.81	3.61E-05
COG1446	B29	0.81	3.61E-05
COG4779	B29	0.81	2.61E-05
COG2356	B29	0.81	2.61E-05
COG3604	B29	0.81	2.61E-05
COG3047	B29	0.81	1.77E-06
COG1261	B29	0.81	1.77E-06
COG1838	B29	0.81	1.77E-06
COG3158	B29	0.81	1.77E-06
COG2360	B29	0.81	1.77E-06
COG2066	B29	0.81	1.77E-06
COG2993	B30	0.85	7.84E-06
COG3278	B30	0.85	7.84E-06
COG0753	B31	0.97	7.69E-06
COG1607	B31	0.97	7.69E-06
COG0114	B31	0.91	1.71E-07
COG0328	B31	0.91	1.71E-07
COG1826	B31	0.91	1.71E-07
COG1651	B31	0.91	1.71E-07
COG1825	B31	0.91	1.71E-07
COG0407	B31	0.83	1.54E-06
COG0746	B31	0.82	1.54E-06
COG0755	B31	0.82	1.54E-06
COG0479	B31	0.82	1.54E-06
COG3717	FAC	0.97	3.95E-05
COG3723	FAJ	0.81	9.22E-05
COG2356	FAL	0.82	5.31E-05
COG0246	FAM	0.85	4.69E-05
COG1175	FAM	0.85	4.69E-05
COG3730	FAP	0.95	2.58E-05
COG3833	FAT	0.94	3.40E-05
COG2182	FAT	0.94	3.40E-05
COG1956	FAT	0.90	4.42E-05
COG3133	FAU	0.85	2.69E-05
COG0643	G03	0.94	4.93E-09
COG1516	G03	0.94	4.93E-09
COG0840	G03	0.94	4.93E-09
COG1345	G03	0.94	4.93E-09
COG1291	G03	0.88	4.19E-08
COG4786	G03	0.88	4.19E-08
COG1677	G03	0.88	4.19E-08
COG1256	G03	0.88	4.19E-08
COG1815	G03	0.88	4.19E-08
COG1558	G03	0.88	4.19E-08
COG1843	G03	0.88	4.19E-08
COG1256	G03	0.88	4.19E-08

COG1360	G03	0.88	4.19E-08
COG1684	G03	0.88	4.19E-08
COG4786	G03	0.88	4.19E-08
COG1815	G03	0.88	4.19E-08
COG1749	G03	0.83	7.02E-07
COG2166	G14	0.85	3.37E-05
