# Supplementary material for: Integration of curated databases to identify genotype-phenotype associations
Source: BMC Genomics. 2006 Oct 12;7:257. doi: 10.1186/1471-2164-7-257 (PMC1630430; doi:10.1186/1471-2164-7-257)
Supplement: Additional file 4 — GIDEON laboratory tests analyzed and their descriptions. [file 1471-2164-7-257-S4.pdf]

Additional Table 4. Gideon Labs Analyzed and their Descriptions

Gideon Lab Abbreviations	Lab Description	Criteria Used to Score Lab as Positive
B01	Gram negative	Gram-negative forms predominate
B02	Gram positive	Gram-positive forms predominate
B28	Growth on ordinary blood agar	Visible growth on sheep blood agar within 48 hours
B29	Growth on MacConkey agar	Visible growth within 48 hours; or within 7 days and non-fermentative gram negative rods
B30	Oxidase	Paper strip test from appropriate media indicate when oxidase is produced
B31	Catalase	Perform on young colonies (up to 24 hours) using 3% hydrogen peroxide. Bubbles indicate a positive reaction (alternative technique for Mycobacteria)
FAC	L-Arabinose	Commercial phenol red techniques indicate when sugar is utilized
FAJ	Lactose	Commercial phenol red techniques indicate when sugar is utilized
FAL	D-Mannitol	Commercial phenol red techniques indicate when sugar is utilized
FAM	D-Mannose	Commercial phenol red techniques indicate when sugar is utilized
FAP	L-Rhamnose	Commercial phenol red techniques indicate when sugar is utilized
FAT	Trehalose	Commercial phenol red techniques indicate when sugar is utilized
FAU	D-Xylose	Commercial phenol red techniques indicate when sugar is utilized
G03	Motile	Standard hanging drop on fresh broth isolates for viewing translational movement
G14	Nitrate to nitrite	Evidence of nitrite production in commercial and self-prepared media
