# Supplementary material for: Integration of curated databases to identify genotype-phenotype associations
Source: BMC Genomics. 2006 Oct 12;7:257. doi: 10.1186/1471-2164-7-257 (PMC1630430; doi:10.1186/1471-2164-7-257)
Supplement: Additional file 5 — COGs analyzed and their descriptions. [file 1471-2164-7-257-S5.pdf]

Additional Table 5. COGs Analyzed and their Descriptions

COG	Protein Name
COG0114	Fumarase
COG0246	Mannitol-1-phosphate/altronate dehydrogenases
COG0328	Ribonuclease HI
COG0407	Uroporphyrinogen-III decarboxylase
COG0408	Coproporphyrinogen III oxidase
COG0417	DNA polymerase elongation subunit (family B)
COG0430	RNA 3-terminal phosphate cyclase
COG0458	Carbamoylphosphate synthase large subunit (split gene in MJ)
COG0479	Succinate dehydrogenase/fumarate reductase, Fe-S protein subunit
COG0643	Chemotaxis protein histidine kinase and related kinases
COG0746	Molybdopterin-guanine dinucleotide biosynthesis protein A
COG0753	Catalase
COG0755	ABC-type transport system involved in cytochrome c biogenesis, permease component
COG0763	Lipid A disaccharide synthetase
COG0774	UDP-3-O-acyl-N-acetylglucosamine deacetylase
COG0805	Sec-independent protein secretion pathway component TatC
COG0811	Biopolymer transport proteins
COG0818	Diacylglycerol kinase
COG0823	Periplasmic component of the Tol biopolymer transport system
COG0835	Chemotaxis signal transduction protein
COG0840	Methyl-accepting chemotaxis protein
COG0848	Biopolymer transport protein
COG1043	Acyl-[acyl carrier protein]--UDP-N-acetylglucosamine O-acyltransferase
COG1044	UDP-3-O-[3-hydroxymyristoyl] glucosamine N-acyltransferase
COG1137	ABC-type (unclassified) transport system, ATPase component
COG1175	ABC-type sugar transport systems, permease components
COG1188	Ribosome-associated heat shock protein implicated in the recycling of the 50S subunit (S4 paralog)
COG1212	CMP-2-keto-3-deoxyoctulosonic acid synthetase
COG1256	Flagellar hook-associated protein
COG1261	Flagellar basal body P-ring biosynthesis protein
COG1291	Flagellar motor component
COG1345	Flagellar capping protein
COG1352	Methylase of chemotaxis methyl-accepting proteins
COG1358	Ribosomal protein HS6-type (S12/L30/L7a)
COG1360	Flagellar motor protein
COG1446	Asparaginase
COG1464	ABC-type metal ion transport system, periplasmic component/surface antigen
COG1495	Disulfide bond formation protein DsbB
COG1516	Flagellin-specific chaperone FliS
COG1519	3-deoxy-D-manno-octulosonic-acid transferase
COG1538	Outer membrane protein
COG1558	Flagellar basal body rod protein
COG1607	Acyl-CoA hydrolase
COG1651	Protein-disulfide isomerase
COG1663	Tetraacyldisaccharide-1-P 4-kinase

COG1668	ABC-type Na+ efflux pump, permease component
COG1677	Flagellar hook-basal body protein
COG1684	Flagellar biosynthesis pathway, component FliR
COG1749	Flagellar hook protein FlgE
COG1815	Flagellar basal body protein
COG1825	Ribosomal protein L25 (general stress protein Ctc)
COG1826	Sec-independent protein secretion pathway components
COG1838	Tartrate dehydratase beta subunit/Fumarate hydratase class I, C-terminal domain
COG1843	Flagellar hook capping protein
COG1953	Cytosine/uracil/thiamine/allantoin permeases
COG1956	GAF domain-containing protein
COG2066	Glutaminase
COG2109	ATP:corrinoid adenosyltransferase
COG2166	SufE protein probably involved in Fe-S center assembly
COG2182	Maltose-binding periplasmic proteins/domains
COG2204	Response regulator containing CheY-like receiver, AAA-type ATPase, and DNA-binding domains
COG2264	Ribosomal protein L11 methylase
COG2344	AT-rich DNA-binding protein
COG2356	Endonuclease I
COG2356	Endonuclease I
COG2360	Leu/Phe-tRNA-protein transferase
COG2747	Negative regulator of flagellin synthesis (anti-sigma28 factor)
COG2877	3-deoxy-D-manno-octulosonic acid (KDO) 8-phosphate synthase
COG2988	Succinylglutamate desuccinylase
COG2993	Cbb3-type cytochrome oxidase, cytochrome c subunit
COG3026	Negative regulator of sigma E activity
COG3047	Outer membrane protein W
COG3073	Negative regulator of sigma E activity
COG3121	P pilus assembly protein, chaperone PapD
COG3130	Ribosome modulation factor
COG3131	Periplasmic glucans biosynthesis protein
COG3133	Outer membrane lipoprotein
COG3137	Putative salt-induced outer membrane protein
COG3143	Chemotaxis protein
COG3158	K+ transporter
COG3160	Regulator of sigma D
COG3166	Tfp pilus assembly protein PilN
COG3248	Nucleoside-binding outer membrane protein
COG3278	Cbb3-type cytochrome oxidase, subunit 1
COG3539	P pilus assembly protein, pilin FimA
COG3599	Cell division initiation protein
COG3604	Transcriptional regulator containing GAF, AAA-type ATPase, and DNA binding domains
COG3717	5-keto 4-deoxyuronate isomerase
COG3723	Recombinational DNA repair protein (RecE pathway)
COG3730	Phosphotransferase system sorbitol-specific component IIC
COG3764	Sortase (surface protein transpeptidase)
COG3833	ABC-type maltose transport systems, permease component
COG3852	Signal transduction histidine kinase, nitrogen specific
COG3966	Protein involved in D-alanine esterification of lipoteichoic acid and wall teichoic acid (D-

	alanine transfer protein)
COG4206	Outer membrane cobalamin receptor protein
COG4238	Murein lipoprotein
COG4775	Outer membrane protein/protective antigen OMA87
COG4779	ABC-type enterobactin transport system, permease component
COG4786	Flagellar basal body rod protein
COG4787	Flagellar basal body rod protein
